# APASL HCV guidelines of virus-eradicated patients by DAA on how to monitor HCC occurrence and HBV reactivation

**DOI:** 10.1007/s12072-019-09988-7

**Published:** 2019-09-20

**Authors:** Tatsuo Kanda, George K. K. Lau, Lai Wei, Mitsuhiko Moriyama, Ming-Lung Yu, Wang-Long Chuang, Alaaeldin Ibrahim, Cosmas Rinaldi Adithya Lesmana, Jose Sollano, Manoj Kumar, Ankur Jindal, Barjesh Chander Sharma, Saeed S. Hamid, A. Kadir Dokmeci, Mamun-Al-Mahtab, Geoffrey W. McCaughan, Jafri Wasim, Darrell H. G. Crawford, Jia-Horng Kao, Yoshihiko Ooka, Osamu Yokosuka, Shiv Kumar Sarin, Masao Omata

**Affiliations:** 1 Division of Gastroenterology and Hepatology, Department of Medicine, Nihon University School of Medicine, Tokyo, Japan; 2 Humanity and Health Medical Center, Hong Kong SAR, China; 3 Tsinghua Changgung Hospital, Tsinghua University, Beijing, China; 4 College of Biological Science and Technology, National Chiao Tung University, Hsin-Chu, Taiwan; 5 Hepatobiliary Division, Department of Internal Medicine, Kaohsiung Medical University Hospital, Kaohsiung Medical University, Kaohsiung, Taiwan; 6 GI/Liver Division, Department of Internal Medicine, University of Benha, Banha, Egypt; 7 Digestive Disease and GI Oncology Centre, Medistra Hospital, Jakarta, Indonesia; 8 Hepatobiliary Division, Department of Internal Medicine, Dr. Cipto Mangunkusumo Hospital, Universitas Indonesia, Jakarta, Indonesia; 9 University Santo Tomas Hospital, Manila, Philippines; 10 Department of Hepatology, Institute of Liver and Biliary Sciences, New Delhi, India; 11 Department of Gastroenterology, G.B. Pant Hospital, New Delhi, India; 12 Department of Medicine, Aga Khan University and Hospital, Stadium Road, Karachi, 74800 Pakistan; 13 Department of Gastroenterology, Ankara University School of Medicine, Ankara, Turkey; 14 Department of Hepatology, Bangabandhu Sheikh Mujib Medical University, Dhaka, 1000 Bangladesh; 15 Royal Prince Alfred Hospital, Centenary Institute, University of Sydney, Sydney, Australia; 16 University of Queensland, School of Medicine, Woolloongabba, QLD 4102 Australia; 17 National Taiwan University College of Medicine, and National Taiwan University Hospital, Taipei, Taiwan; 18 Chiba University, Graduate School of Medicine, Chiba, Japan; 19 Yamanashi Prefectural Central Hospital, 1-1-1 Fujimi, Kofu-shi, Yamanashi, 400-8506 Japan; 20 The University of Tokyo, 7-3-1 Hongo, Bunkyo-ku, Tokyo, 113-8655 Japan

**Keywords:** HCV, HCC, DAA, SVR, Follow-up, Guideline, HBV

## Abstract

In the direct-acting antiviral (DAA) era for hepatitis C virus (HCV) infection, sustained virological response (SVR) is very high, but close attention must be paid to the possible occurrence of hepatocellular carcinoma (HCC) and reactivation of hepatitis B virus (HBV) in patients with co-infection who achieved SVR in short term. HCC occurrence was more often observed in patients *with* previous HCC history. We found occurrence of HCC in 178 (29.6%) of 602 patients *with* previous HCC history (15.4 months mean follow-up post-DAA initiation) but, in contrast, in only 604 (1.3%) of 45,870 patients *without* previous HCC history (18.2 months mean follow-up). Thus, in these guidelines, we recommend the following: in patients *with* previous HCC history, surveillance at 4-month intervals for HCC by ultrasonography (US) and tumor markers should be performed. In patients *without* previous HCC history, surveillance at 6- to 12-month intervals for HCC including US is recommended until the long-term DAA treatment effects, especially for the resolution of liver fibrosis, are confirmed. This guideline also includes recommendations on how to follow-up patients who have been infected with both HCV and HBV. When HCV was eradicated in these HBsAg-positive patients or patients *with* previous HBV infection (anti-HBc and/or anti-HBs-positive), it was shown that HBV reactivation or HBV DNA reappearance was observed in 67 (41.4%) of 162 or 12 (0.9%) of 1317, respectively. For these co-infected patients, careful attention should be paid to HBV reactivation for 24 weeks post-treatment.

**Electronic supplementary material:**

The online version of this article (10.1007/s12072-019-09988-7) contains supplementary material, which is available to authorized users.

## Introduction

Hepatocellular carcinoma (HCC) due to hepatitis C virus (HCV) infection is one of the major causes of liver-related death [1, 2]. Eradication of HCV could reduce the occurrence of HCC, as demonstrated by the long-term follow-up of patients who achieved sustained virological response (SVR) in the interferon era [3–5]. Thus, SVR could be the goal of antiviral therapy for HCV.

In the interferon era, as the duration of interferon-based therapy was longer than that of DAA therapy, the occurrence of HCC has occasionally been observed during the treatment. But these cases were omitted from studies or ignored by regarding them as pre-existing, and they were therefore unrelated to the interferon treatment [5, 6].

Now, in the age of DAAs, extremely high SVR rates, sometimes even 100%, have been reported [7–17]. However, there have been several reports on the unexpectedly high rate of early HCC occurrence despite the virus eradication [18–35]. In real-life settings, if these are actual cases, this incidence could be a shocking event to patients as well to attending physicians. This prompted us to collect data and provide a compact APASL practice guidelines.

In addition, in Asian countries, co-infections of HBV and HCV are more frequently observed. We are likely the first to elucidate the effects of DAA on the replication of HBV by such a high SVR for HCV. Therefore, we have also proposed a compact recommendation on how to follow co-infected patients.

## Part I

### Risk factors for the occurrence of HCC

These days, most patients seen at outpatient clinics are those whose HCV has been eradicated by the use of DAAs. Although more recent clinical studies and real-world studies have reported that DAA therapy decreased the risk of both de novo HCC and recurrent HCC in both cirrhotic and non-cirrhotic patients with HCV infection, several preliminary studies have dealt with the risk factors for the occurrence of HCC [29, 32, 34, 35]. These studies revealed that male gender, older age, alcohol abuse, diabetes mellitus, and the existence of cirrhosis are associated with the occurrence of HCC (Table 1) [29, 32, 34, 35]. Most of these studies were conducted 1–2 years after DAA treatment [11, 33]. Similar factors were also shown to be associated with increased HCC risk during the interferon era [36, 37].

**Table 1:** Risk factors and odds ratio for HCC in direct-acting antiviral (DAA) combination-treated patients [29, 32, 34, 35]

Risk factors for HCC	Odds ratio (95% CI), *n*, *p* value [Refs.]
Cirrhosis	4.73 (3.34–6.68), total *n* = 19,581, HCC (*n*, cirrhosis, yes/no = 139/44), < 0.0001 [29]
Previous HCC history	2.64 (0.90–7.74), total *n* = 864, HCC (*n*, previous HCC history, yes/no = 24/17), 0.075 [32]
Male gender	2.63 (0.65–10), total *n* = 19,581, HCC (*n*, male, yes/no = 181/2), 0.17 [29] 2.09 (0.73–5.98), total *n* = 864, HCC (*n*, male, yes/no = 26/15), 0.167 [32] 1.49 (0.91–2.44), total *n* = 2249, HCC (*n*, male, yes/no = 55/23), 0.11 [35]
Alcohol abuse	1.56 (1.11–2.18), total *n* = 19,581, HCC (*n*, alcohol, yes/no = 124/59), 0.01 [29]
Older age	1.30 (0.96–1.76), total *n* = 19,581, HCC (*n*, >=65, yes/no = 71/112), 0.08 [29]
Diabetes mellitus	1.28 (0.92–1.78), total *n* = 19,581, HCC (*n*, diabetes, yes/no = 96/87), 0.13 [29]
Drug use	1.27 (0.91–1.75), total *n* = 19,581, HCC (*n*, drug, yes/no = 91/92), 0.15 [29]
Bilirubin	1.25 (0.97–1.62), total *n* = 2249, HCC (*n* = 78), 0.08 [35]
Low albumin	1.92 (1.16–3.22), total *n* = 2249, HCC (*n* = 78), 0.010 [35]
EOT-AFP (=>9 ng/mL)	1.19 (1.07–1.34), total *n* = 1523, HCC (*n* = 20), 0.0027 [34]
Low platelet count	1.01 (1.01–1.02), total *n* = 2249, HCC (*n* = 78), 0.011 [35]

*AFP* α-fetoprotein, *EOT* end of treatment, *n* number

However, in addition to the abovementioned parameters, there is some dispute regarding the difference in occurrence of HCC between patients *with* and *without* previous HCC history [19–21, 38]. Thus, we conducted a literature search, investigating the occurrence of HCC in DAA-treated patients *with* and *without* previous HCC history (Table 2) [18–35]. A summary of the collected data is described in the following sections.

**Table 2:** Occurrence of hepatocellular carcinoma (HCC) in patients with direct-acting antiviral (DAA) treatment and sustained virological response (SVR) [18–35]

Authors (year) [references]	Total SVR patients (*n*)	Observation periods (mean months post-DAA initiation)	Patients with HCC occurrence [*n* (%)]	Annual incidence of HCC (%/year)
Minami et al. (2016) [18]	22	5.8	4 (18)	37.2
Reig et al. (2016) [19]	58	5.7	16 (27.6)	58.1
Torres et al. (2016) [20]	84	12	0 (0)	0
Conti et al. (2016) [21]	403	9	26 (6.5)	8.7
Kolly et al. (2017) [22]	47	12	27 (57.4)	57.4
Cardoso et al. (2017) [23]	54	18	4 (7.4)	4.9
Calleja et al. (2017) [24]	70	12	21 (30)	30
Ikeda et al. (2017) [25]	155	12	47 (30.2)	30.2
Mettke et al. (2017) [26]	158	17.5	6 (3.8)	2.61
Nakao et al. (2017) [27]	242	6	6 (2.5)	5.0
Nagata et al. (2017) [28]	729	24.6	29 (4.0)	1.95
Kanwal et al. (2017) [29]	19,518	15.8	183 (0.9)	0.68
Ioannou et al. (2017) [30]	19,909	18	280 (1.4)	0.93
Cabibbo et al. (2018) [31]	143	12	24 (16.8)	16.8
Ooka et al. (2018) [32]	864	15	41 (4.7)	3.76
Reddy et al. (2018) [33]	893	36	16 (1.8)	0.60
Ogawa et al. (2018) [34]	1675	17	46 (2.7)	1.91
Calvaruso et al. (2018) [35]	2140	14	64 (3.0)	2.57
**Total**	**47,164**	**14.6 (5.7–36)**	**840 (1.8)**	**14.6 (0–58.1)**

*n* number

### Occurrence of HCC in patients *without* previous HCC history

The occurrence of HCC after SVR in patients *without* previous HCC history was reported in ten studies (Table 3). In those 10 studies, the total number of SVR patients ranged from 54 to 19,909 patients (mean: 4587 patients). The mean follow-up period of those studies was 18.2 months (range 9–36 months) post-DAA initiation. The overall occurrence rate of HCC after SVR in 45,870 patients *without* previous HCC history was 604 (1.3%) (range 0.9–7.4%) (Table 3) [21, 23, 26, 28–30, 32–35]. Thus, the annual occurrence rate of SVR patients by DAA *without* previous HCC history is no different from that of the interferon era [3–5, 39–41].

**Table 3:** Occurrence of hepatocellular carcinoma (HCC) after direct-acting antiviral (DAA) treatment and sustained virological response (SVR) in patients *without* previous HCC history [21, 23, 26, 28–30, 32–35]

Authors (year) [references]	Total SVR patients (*n*)	Observation periods (months post-DAA initiation)	Patients with HCC occurrence [*n* (%)]	Annual incidence of HCC (%/year)
Conti et al. (2016) [21]	254	9	7 (2.7)	3.60
Cardoso et al. (2017) [23]	54	18	4 (7.4)	4.93
Mettke et al. (2017) [26]	158	17.5	6 (3.8)	2.61
Nagata et al. (2017) [28]	652	21.6	7 (1.1)	0.61
Kanwal et al. (2017) [29]	19,518	15.8	183 (0.9)	0.68
Ioannou et al. (2017) [30]	19,909	18	280 (1.4)	0.93
Ooka et al. (2018) [32]	769	15	17 (2.2)	1.76
Reddy et al. (2018) [33]	893	36	16 (1.8)	0.60
Ogawa et al. (2018) [34]	1523	17	20 (1.3)	0.92
Calvaruso et al. (2018) [35]	2140	14	64 (3.0)	2.57
**Total**	**45,870**	**18.2 (9–36)**	**604 (1.3)**	**1.92 (0.60–4.93)**

*n* number

Therefore, the same guidelines and recommendations as were present in the time of interferon may apply to patients treated by DAAs, if there is no previous experience of HCC. Of course, regular follow-ups are necessary, according to the routinely set rules of the interferon era, especially among HCV patients with advanced liver fibrosis or cirrhosis (Table 3) [42, 43].

### Occurrence of HCC in patients *with* previous HCC history

The occurrence of HCC after DAA treatment and SVR in patients *with* previous HCC history was reported in six studies (Table 4) [21, 24, 25, 28, 32, 34]. The total number of SVR patients ranged from 53 to 155 patients (mean: 100 patients). The mean follow-up period of these studies was 15.4 months (range 9–28 months) post-DAA initiation. The overall occurrence rate of HCC after DAA treatment and SVR in patients *with* previous HCC history was 29.6% (178/602) (range 17.1–71.6%) (Table 4) [21, 24, 25, 28, 32, 34].

**Table 4:** Occurrence of hepatocellular carcinoma (HCC) after direct-acting antiviral (DAA) treatment and sustained virological response (SVR) in patients *with* previous HCC history [21, 24, 25, 28, 32, 34]

Authors (year) [references]	Total SVR patients (*n*)	Observation periods (months post-DAA initiation)	Patients with HCC occurrence [*n* (%)]	Annual incidence of HCC (%/year)
Conti et al. (2016) [21]	53	9	38 (71.6)	95.5
Calleja et al. (2017) [24]	70	12	21 (30)	30.0
Nagata et al. (2017) [28]	77	27.6	22 (28.6)	12.4
Ikeda et al. (2017) [25]	155	12	47 (30.3)	30.3
Ooka et al. (2018) [32]	95	15	24 (25.3)	20.2
Ogawa et al. (2018) [34]	152	17	26 (17.1)	12.1
**Total**	**602**	**15.4 (9–27.6)**	**178 (29.6)**	**33.4 (12.1–95.5)**

*n* number

The very high incidence of HCC occurrence during and right after DAA treatment suggests that very careful attention should be paid to the possible occurrence of HCC in patients *with* previous HCC history.

## Discussion

### Risk of HCC occurrence among patients post-DAA treatment

In the interferon era, male gender, older age, and the existence of cirrhosis and other factors were shown to be associated with risk factors of HCC occurrence [42, 44, 45]. Also in the age of DAAs, similar factors are shown to be associated with this risk. In other words, the existence of cirrhosis, no SVR, male gender, alcohol abuse, older age, and diabetes mellitus are risk factors for HCC occurrence (Table 1) [29, 32, 34, 35]. Surveillance is recommended for SVR patients with any histologic stage of HCV with comorbidities, such as alcohol abuse and diabetes mellitus [1].

Of note, most importantly, the current survey revealed that the existence of previous HCC history is an independent, very high-risk factor for HCC occurrence post-DAA treatment.

In the interferon era, because the treatment duration was longer than that of DAA, several studies seemed to exclude HCC occurrence during and right after interferon treatment when analyzing their data. In fact, during the interferon era, many patients with HCC or cirrhosis could not receive interferon treatment. To some extent, this might explain the lower occurrence of HCC during or right after antiviral treatment.

It has been reported that several mechanisms may exist during and after DAA treatment, such as rapid immunological changes, that could lead to HCC occurrence [46–50]. Changes in cytokines and chemokines have been observed in HCC occurrence post-DAA treatment and it is possible that they may have affected tumor immunity [46–50].

DAA treatment increased the serum vascular endothelial growth factor (VEGF) level which is significantly related to the serum angiopoietin-2 level. These are risk factors for HCC occurrence post-DAA treatment [51, 52]. Rapid immunological changes, including in NKG2D systems, are also observed during and after DAA treatment [53, 54].

With such drastic “environmental changes” occurring in the liver due to the very powerful DAAs, pre-existing “occult neoplastic” or “dysplastic” cells may develop into classical tumors in a short time period. Ooka et al. reported that “dysplastic” nodules detected by ultrasonography (US) might turn into hyper-vascular “classical” HCC by rapid decrease of the immune surveillance system with rapid elimination of HCV [32, 46, 55]. Studies have proposed that the presence of “dysplastic” nodules by US has a much higher odds ratio (26 times) than previous HCC history [32, 56].

In fact, approximately 50% of HCC occurrence and recurrence cases are observed during and 1–2 years after DAA treatment [21, 33]. Although it is well known that patients with mild/no fibrosis and SVR have a lower risk of developing HCC, population-based studies were different from clinical practice guidelines. So, we recommend that, for patients with SVR and risk factors of HCC, surveillance for HCC should be conducted at shorter intervals, and especially within 2 years post-DAA treatment.

How to follow these patients? Among imaging modalities (US, CT, and MRI), US might be the most cost-effective and easily available modality.

The prognosis of HCC depends on earlier-stage detection and earlier treatment [1]. In addition to US, measurement of tumor markers may play a more important role in HCC screening. Among tumor markers for the diagnosis of HCC, measurement of AFP has been performed for decades [57, 58], although with some dispute regarding its significance. However, there have been numerous studies regarding multiple tests including lens culinaris agglutinin (LCA)-reactive AFP isoform (AFP-L3), which can differentiate an increase in AFP due to HCC from that in patients with benign liver disease. In addition, des-γ-carboxy prothrombin (DCP) is a very powerful measure for detecting early and small tumors [59–65]. These studies are mostly from Japan, and these two tests, AFP-L3 and DCP, could not be validated as they have not been available in many countries. However, AFP, AFP-L3, and DCP tests have now become increasingly available in many Asian countries. Thus, we recommended the measurements of these markers.

Once a blood test result is abnormal, further imaging modalities [gadolinium ethoxybenzyl diethylenetriamine pentaacetic acid (Gd-EOB-DTPA)-enhanced MRI and/or dynamic CT] should be performed for the potential diagnosis of HCC occurrence [1, 32].

Thus, in patients *with* previous HCC history, surveillance of shorter 4-month intervals for HCC, including US with AFP, AFP-L3, and/or DCP, should be performed [1].

After successful eradication of HCV, regular follow-up of HCC, esophageal varices, and other complications of advanced liver fibrosis will be necessary if they existed at pre-treatment [66–68].

### #1 Consensus statements and recommendations on follow-up of DAA-treated virus-eradicated HCV-infected patients

1. In patients *without* advanced liver fibrosis, or cirrhosis and *without* previous HCC history
  1. Before, during, and approximately 2 years after the end of treatment (EOT) with DAA, surveillance at 6-month intervals for HCC, including ultrasonography (US) with or without tumor markers, should be performed (*C*-*2*).
  2. After 2 years, surveillance at 12-month intervals for HCC, including US with or without AFP, could be performed (*C*-*2*).
2. In patients with advanced liver fibrosis or cirrhosis and *without* previous HCC history
  Surveillance at 6-month intervals for HCC, including by US with AFP, lens culinaris agglutinin (LCA)-reactive AFP isoform (AFP-L3) and/or des-γ-carboxy prothrombin (DCP) should be performed (*A*-*1*).
3. In patients *with* previous HCC history
  Surveillance at 4-month intervals for HCC, including by US with AFP, AFP-L3 and/or DCP, should be performed. In these cases, contrast-enhanced US (CEUS), dynamic CT, dynamic MRI or gadolinium ethoxybenzyl diethylenetriamine pentaacetic acid (Gd-EOB-DTPA)-enhanced MRI could be added (*A*-*2*).
4. SVR patients with alcohol abuse and/or diabetes mellitus should undergo surveillance for HCC regularly (*A*-*1*).
5. In patients *with* advanced liver fibrosis or cirrhosis, screening for esophageal and gastric varices by endoscopy should be performed especially if present at pre-treatment (*A*-*1*).

Grading of evidence and recommendations are shown in Supplementary Table 1.

## Part II

### HBV reactivation in patients with HCV and HBV co-infection

HBV infection is one of the major health problems in the world, with the highest rates being in Africa and the Asia–Pacific region [69]. Evaluation for HBV infection was also recommended for all persons with active HCV infection by the US Food and Drug Administration in 2004. However, the exact prevalence and characteristics of HBV DNA reappearance and clinical “reactivation” among patients treated by DAAs are not known in detail.

Therefore, we collected data from 14 studies on HBV DNA reappearance and clinical reactivation in HBV and HCV co-infected patients treated by DAAs (Table 5) [70–78].

**Table 5:** Hepatitis B virus (HBV) reactivation or HBV DNA reappearance in patients with HBV and hepatitis C virus (HCV) co-infection after direct-acting antiviral (DAA) treatment [70–78]

Authors (year) [references]	Total patients (*n*)	Observation periods (months post-EOT)	Patients with increases of HBV DNA greater than 1 log10 IU/mL or HBV DNA reappearance [*n* (%)]	Monthly incidence of HBV reactivation or HBV DNA reappearance (%/month)
*HBsAg*-*positive patients*				
Gane et al. (2016) [70]	8	3	7 (87.5)	29.2
Doi et al. (2017) [71]	4	3	2 (50)	16.7
Kawagishi et al. (2017) [72]	4	3	2 (50)	16.7
Yeh et al. (2017) [73]	7	3	7 (100)^e^	33.3
Mucke et al. (2017) [74]	8	3	4 (50)^b^	16.7
Wang et al. (2017) [75]	10	3	3 (33.3)^d^	11.1
Tamori et al. (2018) [76]	12	3	3 (25)^c^	8.3
Liu et al. (2018) [77]	109	3	39 (35.8)^a^	13
**Total**	**162**	**3**	**67 (41.4)**	**18.1 (8.33–33.3)**
*HBsAg*-*negative patients positive for anti*-*HBc antibody and/or anti*-*HBs antibody*				
Yeh et al. (2017) [73]	57	3	0 (0)	0
Wang et al. (2017) [75]	124	3	0 (0)	0
Doi et al. (2017) [71]	155	3	3 (1.9)	0.63
Kawagishi et al. (2017) [72]	153	3	4 (2.6)	0.87
Ogawa et al. (2018) [78]	63	3	4 (6.3)	2.1
Tamori et al. (2018) [76]	765	3	1 (0.1)	0.33
**Total**	**1317**	**3**	**12 (0.91)**	**0.61 (0–2.1)**

*HBsAg* hepatitis B surface antigen, *anti-HBc* anti-hepatitis B core antibody, *anti-HBs* anti-hepatitis B surface antibody, *EOT* end of treatment, *n* number

^a^Three patients (one with cirrhosis and two without cirrhosis) began anti-HBV treatment: one entecavir (ETV) and two tenofovir disoproxil fumarate (TDF)

^b^Three patients (one with cirrhosis and two without cirrhosis) began TDF

^c^One cirrhotic patient began TDF

^d^Two patients, one had hepatic failure and one had icteric hepatitis

^e^One icteric patient began ETV

#### HBsAg-positive group

Of these 14 studies, 8 reported the results of HBV DNA reappearance and clinical reactivation in HBsAg-positive patients treated by DAAs (Table 5) [70–77]. The number of patients enrolled in those 8 studies ranged from 4 to 109 patients (mean 20) and the mean observation period was 3 months post-EOT. The overall occurrence rate of HBV DNA reappearance and clinical reactivation among 162 patients treated by DAA was 41.4% (67/162) (range 25–100%). Thus, among HBsAg-positive patients, HBV DNA reappearance and reactivation are the frequent events through at least 12 weeks after EOT (Table 5).

#### HBsAg-negative group (anti-HBc- and/or anti-HBs-positive group)

Of the 14 studies (Table 5), 6 studies reported results on HBV DNA reappearance and clinical reactivation in HBsAg-negative, but positivity for anti-hepatitis B core (anti-HBc) antibody and/or anti-hepatitis B surface (anti-HBs) antibody at baseline [71–73, 75, 76, 78].

The number of patients enrolled in those 6 studies ranged from 57 to 765 patients (mean 219.5) and the mean observation period was 3 months post-EOT. The overall occurrence rate of HBV DNA reappearance and clinical reactivation among 1317 patients treated by DAAs was 0.91% (12/1317) (range 0–6.3%) (Table 5). Thus, in HBsAg-negative patients but positive for anti-HBc antibody and/or anti-HBs antibody at baseline; HBV reactivation and/or HBV DNA reappearance are rare events through 12 weeks after EOT (Table 5).

## Discussion

### Clinical pictures of HBV reactivation

#### HBsAg-positive group

Before the rituximab (humanized anti-CD20 monoclonal antibody) era, Lau et al. reported that among 15 HBsAg-positive patients with lymphoma treated with chemotherapy but deferred prophylactic lamivudine therapy, 8 (53%) had HBV reactivation defined as an increase of serum HBV DNA to more than 10 times of baseline [79]. Of these eight patients, seven (87.5%) had ‘hepatitis’, defined as a more than threefold increase of serum ALT on two consecutive determinations at least 5 days apart. Of these seven patients, anicteric and icteric hepatitis and hepatic failure were 5, 1, and 1, respectively [79]. Thus, of HBsAg-positive patients with lymphoma treated by chemotherapy without nucleos(t)ide analogs, 6.7% (1/15) had hepatic failure [79]. They also observed that, among 15 patients with lymphoma who received lamivudine 1 week before chemotherapy, none had HBV reactivation after chemotherapy [79]. Lok et al. also observed 18 HBV reactivations (67%) [6 icteric hepatitis (22%); 1 non-fatal hepatic failure (3.7%); and 1 death (3.7%)] among 27 Chinese patients who underwent induction cytotoxic therapy without prophylaxis for HBsAg-positive malignant lymphoma [80].

After rituximab was introduced as a potent drug for patients with malignant lymphoma, reactivation of HBV has been repeatedly shown in HBsAg-positive patients [81]. Wang et al. reported that rituximab/chemotherapy induced hepatic dysfunction in 13 (33%) of 40 HBsAg-positive patients with diffuse large B cell lymphoma [82].

HBV reactivation has also been reported in HBsAg-positive solid cancer patients who underwent chemotherapy or other molecular target therapies [69]. Among HBsAg-positive breast cancer patients receiving chemotherapy, the rates of HBV reactivation in patients without or with prophylactic lamivudine were 28.6% and 0%, respectively [83]. HBV reactivation during chemotherapy occurred independently of lymphoma (odds ratio: 5.0), breast cancer (odds ratio: 4.2), steroid use (odds ratio: 2.7), and HBV DNA positive at baseline (odds ratio: 8.4) [84].

Thus, APASL HBV guidelines have recommended that prophylactic nucleos(t)ide therapy should be given to HBsAg-positive cancer patients who receive cytotoxic and immunosuppressive therapy, regardless of HBV DNA levels for 12 months after cessation [69].

Regarding the treatment by DAAs for those co-infected with HBV and HCV, a variety of events, ranging from asymptomatic HBV reactivation/HBV DNA reappearance to clinically symptomatic reactivation characterized by elevation in HBV DNA and ALT were reported [70–77].

Collected results of eight studies of HBsAg-positive and co-infected patients treated with DAAs for HCV infection indicated that the rates of HBV reactivation were similar to HBsAg-positive patients with malignant lymphoma and cancer patients treated with cytotoxic drugs (~ 40%) [85, 86] (also see Table 5).

Regarding the severity of liver disease induced by this HBV reactivation in HBsAg-positive patients treated with DAAs, only limited data are available [75, 77, 87–89]. Bersoff-Matcha et al. reported that two cases with liver failure resulted in one death and one case of liver transplantation [87]. Wang et al. also reported one HBsAg-positive patient with liver failure due to HBV reactivation although most reported cases were asymptomatic increases of HBV DNA and/or ALT in the absence of concomitant liver injury [75].

Of note, Liu et al. reported that two patients had concomitant elevation of HBV DNA level with ALT elevation > 2 times the upper limit of normal at post-treatment week 48, of whom one commenced treatment with entecavir at post-treatment week 53 following the onset of malaise, anorexia, and nausea associated with sclera jaundice [77]. Holmes et al. reviewed two HBsAg-positive, co-infected patients who were treated by DAAs for HCV infection, and had fulminant hepatic failure and death: one was a 57-year-old female who was treated with daclatasvir plus asunaprevir, had HBV reactivation at week 8 from the start of DAAs, and she was treated with entecavir; the other was a 73-year-old female who was treated with daclatasvir plus asunaprevir, had HBV reactivation at week 7 from the start of DAAs, and she had stopped entecavir prior to the commencement of DAA therapy [88].

At present, we do not know the risk factors of HBV reactivation and associated liver failure, although several factors such as HBsAg levels and HBV DNA levels have been reported [76, 90].

For the safety of HBsAg-positive patients treated by DAAs, we recommend that prophylactic nucleos(t)ide therapy should be given before starting DAA therapy; nonetheless, further studies may also be needed to determine the duration of prophylactic nucleos(t)ide therapy.

#### HBsAg-negative, but positive for anti-HBc and/or anti-HBs group

Although the elimination of HBsAg is one of the goals in the treatment of HBV infection, HBV DNA reappeared in 15–33% patients after HBsAg seroclearance in the natural history of HBV infection and in post-anti-HBV treatment [91]. So, it is possible that HBV reactivation and/or HBV DNA reappearance may occur in patients of this group treated by DAAs, as well as patients who receive immunosuppressants or anti-cancer drugs (Table 6).

**Table 6:** HBV reactivation in HBsAg-negative patients treated for lymphoma and solid tumors

Types	Prophylactic nucleos(t)ide analogs	Total patients (*n*)	Incidence [*n*, (%)]	Authors (year) [references]
Lymphoma (without rituximab-based regimens)	NA	72	10 (14%)	Lok et al. (1991) [80]
Lymphoma (with rituximab-based regimens)	NA	39	7 (17.9%)	Huang et al. (2013) [93]
Hematologic malignancy (with rituximab-based regimens)	NA	28	3 (10.7%)	Buti et al. (2014) [94]
Lymphoma (with rituximab-based regimens)	NA	578	36 (6.3%)	Mozessohn et al. (2015) [92]
Solid cancer	NA	27	2 (7.4%)	Hagiwara et al. (2012) [98].
Solid cancer	NA	321	1 (0.3%)	Kim et al. (2014) [97]

*NA* not applicable

With the administration of rituximab without antiviral treatment, clinical HBV reactivation was estimated at 6.3% in HBsAg-negative/anti-HBc-positive patients with lymphoma [92]. Prior to use of rituximab, Lok et al. also observed 10 HBV reactivations (14%) [1 icteric hepatitis (2%); 1 non-fatal hepatic failure (2%); and no death (0%)] of 72 HBsAg-negative patients with malignant lymphoma treated by chemotherapy without prophylactic treatment of nucleos(t)ide analogs (Table 6) [80].

Thus, in the rituximab era, once HBsAg-negative patients who received rituximab including chemotherapy for malignant lymphoma had HBV reactivation (6.3–17.9%) (Table 6) [92–94], higher mortality rates (12.5–50%) were seen [95].

In breast cancer, HBV fetal reactivation was occasionally observed in HBsAg-negative patients who underwent chemotherapy [96]. Kim et al. reported that HBV reactivation occurred in 1 (0.3%) of 321 HBsAg-negative and anti-HBc-positive patients with solid cancers during anti-cancer chemotherapy [97]. Hagiwara et al. reported that HBV reactivation occurred in 2 (7.4%) of 27 HBsAg-negative and anti-HBc/anti-HBs-positive patients with solid cancers during anti-cancer chemotherapy [98].

Jun et al. reported that 2 (10%), 8 (5.3%), 4 (5.5%), and 2 (0.9%) HBV reactivations were observed in 20 HBsAg(−)/anti-HBc(+)/anti-HBs(−), 151 HBsAg(−)/anti-HBc(+)/anti-HBs(+), 73 HBsAg(−)/anti-HBc(−)/anti-HBs(−), and 227 HBsAg(−)/anti-HBc(−)/anti-HBs(+) patients undergoing hematopoietic stem cell transplantation, respectively [99]. Of note, the incidence of HBV reactivation in these HBsAg-negative patients was not low (5.9%) [99, 100], although most patients with solid cancers remained unscreened for HBV-resolved infection [101, 102].

A summary of six studies of HBsAg-negative cases indicates that the overall occurrence rate of HBV reactivation and/or HBV DNA reappearance is lower (0.91%) (Table 5).

The prevalence rates of HBV reactivation and/or HBV DNA reappearance in patients of the HBsAg-negative/anti-HBc-positive group by DAAs seem equal to or less than those with chemotherapy for breast cancer, one of the non-hematologic malignancies.

Regarding the severity of liver disease induced by this HBV reactivation in HBsAg-negative patients treated by DAA, only limited data are available [103, 104]. Two HBsAg-negative patients who developed hepatic failure after DAA treatment have been reported (Table 7) [103, 104]. We do not know the exact risk factors of HBV reactivation in HBsAg-negative patients treated with DAAs although several factors have been reported [71–73, 75, 76, 78].

**Table 7:** Cases with HBV reactivation-related hepatic failure among co-infected patients treated by DAAs

#	Age (years)/gender	Treatment for HCV (GT)	Severity, ALT levels	Treatment for HBV (GT/HBeAg)/outcome	Authors (year) [references]
HBsAg-positive patients treated by DAAs					
1	57/Female	Daclatasvir/Asunaprevir (unknown)	Hepatic failure, ALT 2114 IU/L	Entecavir (unknown/unknown)/death	Holmes et al. (2017) [88]
2	73/Female	Daclatasvir/Asunaprevir (unknown)	Hepatic failure, ALT 462 IU/L	Entecavir (unknown/unknown)/death	Holmes et al. (2017) [88]
3	53/Female	Sofosbuvir/Ribavirin (GT1)	ALT 1417 IU/L	No description (unknown/HBeAg-)/no description	Holmes et al. (2017) [88]
4	53/Male	Ledipasvir/Sofosbuvir (GT1) [co-infection with HIV]	ALT 1026 IU/L	Tenofovir (GTD/HBeAg-)/alive	De Monte et al. (2016) [89]
HBsAg-negative patients treated by DAAs					
5	59/Female	Sofosbuvir/Simeprevir (GT1b)	Hepatic failure, ALT 2263 IU/L	Tenofovir (unknown/unknown)/liver transplantation	Ende et al. (2015) [103]
6	83/Female	Daclatasvir/Asunaprevir (GT1b)	Hepatic failure, ALT 1066 IU/L	Entecavir (GTB1/unknown)/death	Hayashi et al. (2016) [104]

*GT* genotype, *ALT* alanine aminotransferase, *HBeAg* hepatitis B e antigen

There are no standard management regimens for HBV reactivation among HBsAg-negative patients, even for those treated with rituximab including chemotherapy. It has been reported that monthly monitoring of HBV DNA is useful for preventing HBV reactivation-related hepatitis among B cell non-Hodgkin lymphoma patients with resolved HBV infection following rituximab plus corticosteroid including chemotherapy [105].

Physicians should be aware of the risk of HBV reactivation in HBsAg-negative patients. Although further studies are needed to compare the efficacy and cost effectiveness of different preventive strategies, we should at least perform careful monitoring of these patients, and if needed, we should administer nucleos(t)ide analogs against HBV DNA reactivation/reappearance. Regarding nucleos(t)ide analogs, as lamivudine and telbivudine are limited, entecavir or tenofovir would be preferred.

### #2 Consensus statements and recommendations on follow-up of HBV and HCV co-infected patients treated with DAA in Asia–Pacific region

1. Before starting DAA treatment, HBsAg should be examined in high endemic areas of HBV infection *(A*-*1)*.
2. In HBsAg-positive patients *with* advanced fibrosis, cirrhosis or previous HCC, pre-emptive nucleos(t)ide analog treatment should be started to prevent HBV reactivation *(A*-*1)*.
3. In HBsAg-positive patients *without* advanced fibrosis, cirrhosis or previous HCC history, pre-emptive nucleos(t)ide analog treatment is effective for HBV infection *(A*-*1)*, or close monitoring should be recommended during DAA treatment and through 24 weeks after EOT *(B*-*1)*. Stopping should follow APASL HBV guidelines.
4. In HBsAg-negative patients who are positive for anti-HBc antibody and/or anti-HBs antibody when abnormal liver function tests are observed during DAA treatment and after EOT, HCV RNA, HBsAg and HBV DNA should be examined. Nucleos(t)ide analogs should be used to treat HBV reactivation *(B*-*1)*.

Grading of evidence and recommendations are shown in Supplementary Table 1.

## Conclusion

**Fig. 1:**
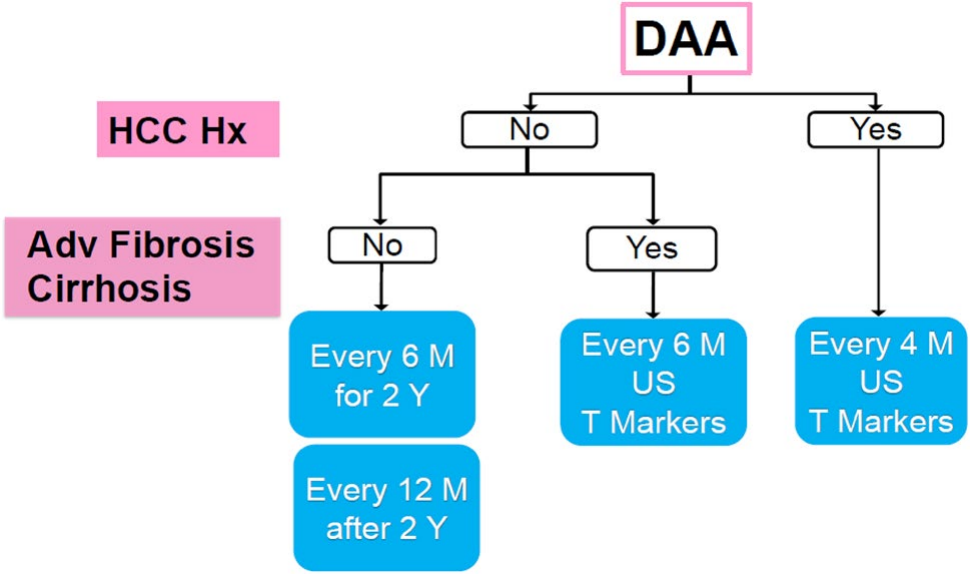
Surveillance/monitoring algorithm for patients with hepatitis C virus and sustained virological response by direct-acting antivirals (DAAs). *HCC Hx* history of hepatocellular carcinoma, *Adv Fibrosis* advanced liver fibrosis, *US* ultrasonography, *T* Markers: α-fetoprotein (AFP), lens culinaris agglutinin (LCA)-reactive AFP isoform (AFP-L3) and/or des-γ-carboxy prothrombin (DCP)

**Fig. 2:**
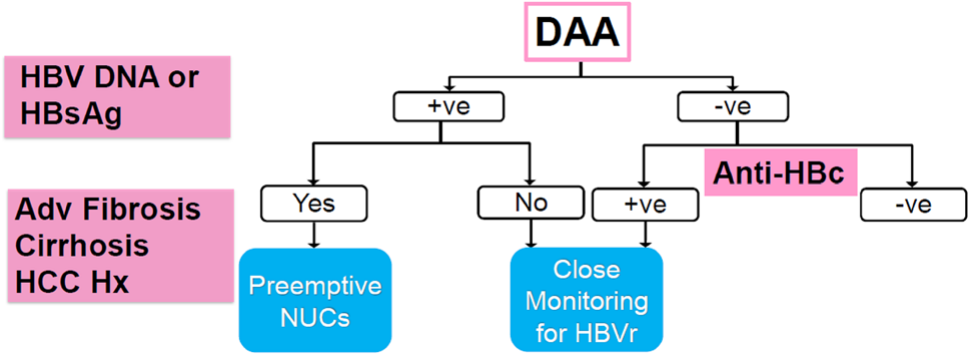
Surveillance/monitoring algorithm for patients co-infected with hepatitis C virus and hepatitis B virus (HBV) and treated with direct-acting antivirals (DAAs). *HCC Hx* history of hepatocellular carcinoma, *Adv Fibrosis* advanced liver fibrosis, *NUCs* nucleos(t)ides, *HBsAg* hepatitis B virus surface antigen, *anti-HBc* ant-hepatitis B virus core antibody, *HBVr* HBV reactivation and/or HBV DNA reappearance, *+ve* positive, *-ve* negative

During DAA treatment, host immunological changes may occur although DAA treatment can lead to higher SVR rates with shorter treatment duration and less serious adverse events in most patients infected with HCV [10]. First, we have created guidelines for the monitoring of HCC occurrence based on its accumulated data for it (Fig. 1). Second, we have constructed compact guidelines for patients with HBsAg and anti-HBc and/or anti-HBs antibody (Fig. 2).

## Electronic supplementary material

Below is the link to the electronic supplementary material.

Supplementary material 1 (DOCX 11 kb)

## Abbreviations

HCV: Hepatitis C virus
HBV: Hepatitis B virus
GT: Genotype
HCC: Hepatocellular carcinoma
DAAs: Direct-acting antivirals
SVR: Sustained virological response
EOT: End of treatment
US: Ultrasonography
AFP: α-Fetoprotein
AFP-L3: Lens culinaris agglutinin (LCA)-reactive AFP isoform
DCP: Des-γ-carboxy prothrombin
