# Supplementary material for: APASL HCV guidelines of virus-eradicated patients by DAA on how to monitor HCC occurrence and HBV reactivation
Source: Hepatol Int. 2019 Sep 20;13(6):649–61. doi: 10.1007/s12072-019-09988-7 (PMC6861433; doi:10.1007/s12072-019-09988-7)
Supplement: Supplementary file 1 — Supplementary material 1 (DOCX 11 kb) [file 12072_2019_9988_MOESM1_ESM.docx]

**Suppl. Table 1.** Grading of evidence and recommendations (adapted from the GRADE system) [1, 2].

| **Grading of evidence** | **Notes** | **Symbol** |
| --- | --- | --- |
| High quality | Further research is very unlikely to change our confidence in the estimate of the effect. | A |
| Moderate quality | Further research is likely to have an important impact on our confidence in the estimate of the effect and may change the estimate. | B |
| Low or very low quality | Further research is very likely to have an important impact on our confidence in the estimate of the effect and is likely to change the estimate. Any estimate of the effect is uncertain. | C |
| **Grading of recommendation** | **Notes** | **Symbol** |
| Strong recommendation warranted | Factors influencing the strength of the recommendation included the quality of the evidence, presumed important patient outcomes, and cost. | 1 |
| Weaker recommendation | Variability in preferences and values or more uncertainty make it more likely that a weak recommendation is warranted. Recommendation is made with less certainty; higher cost or resource consumption. | 2 |

References

1. Guyatt GH, Oxman AD, Vist GE, Kunz R, Falck-Ytter Y, Alonso-Coello P, et al. GRADE: an emerging consensus on rating quality of evidence and strength of recommendations. BMJ 2008;336:924–926

2. Schunemann HJ, Oxman AD, Brozek J, Glasziou P, Jaeschke R, Vist GE, et al. Grading quality of evidence and strength of recommendations for diagnostic tests and strategies. BMJ 2008;336:1106–1110
